# Higher Nucleoporin-Importinβ Affinity at the Nuclear Basket Increases Nucleocytoplasmic Import

**DOI:** 10.1371/journal.pone.0081741

**Published:** 2013-11-25

**Authors:** Mohammad Azimi, Mohammad R. K. Mofrad

**Affiliations:** Molecular Cell Biomechanics Laboratory, Departments of Bioengineering and Mechanical Engineering, University of California, Berkeley, California, United States; Università degli Studi di Milano, Italy

## Abstract

Several *in vitro* studies have shown the presence of an affinity gradient in nuclear pore complex proteins for the import receptor Importinβ, at least partially contributing to nucleocytoplasmic transport, while others have historically argued against the presence of such a gradient. Nonetheless, the existence of an affinity gradient has remained an uncharacterized contributing factor. To shed light on the affinity gradient theory and better characterize how the existence of such an affinity gradient between the nuclear pore and the import receptor may influence the nucleocytoplasmic traffic, we have developed a general-purpose agent based modeling (ABM) framework that features a new method for relating rate constants to molecular binding and unbinding probabilities, and used our ABM approach to quantify the effects of a wide range of forward and reverse nucleoporin-Importinβ affinity gradients. Our results indicate that transport through the nuclear pore complex is maximized with an effective macroscopic affinity gradient of 2000 µM, 200 µM and 10 µM in the cytoplasmic, central channel and nuclear basket respectively. The transport rate at this gradient is approximately 10% higher than the transport rate for a comparable pore lacking any affinity gradient, which has a peak transport rate when all nucleoporins have an affinity of 200 µM for Importinβ. Furthermore, this optimal ratio of affinity gradients is representative of the ratio of affinities reported for the yeast nuclear pore complex – suggesting that the affinity gradient seen *in vitro* is highly optimized.

## Introduction

### The Nuclear Pore Complex and Nucleocytoplasmic Transport

Spatial segregation of genetic material in the nucleus from the cytoplasm gives eukaryotes the ability to highly regulate gene expression and DNA replication. By regulating import to and export from the nucleus, the nuclear pore complex (NPC) plays a critical role in cell physiology – enabling rapid yet selective bi-directional flow of material into and out of the nucleus to maintain cellular functions. For example, RNA synthesized in the nucleus must be shuttled to the cytoplasm for protein synthesis while proteins involved in the transcription of these RNAs must simultaneously be shuttled into the nucleus. While small molecules (Stoke's radius less than ∼2.5 nm) passively diffuse across the channel, larger molecules rely on an active (energy dependent) mechanism for transport. This pore's selectivity for active cargo transport is achieved through a combination of structural features and biochemical pathways that lead to a still elusive transport mechanism for which there exist several competing hypotheses.

**Figure 1:**
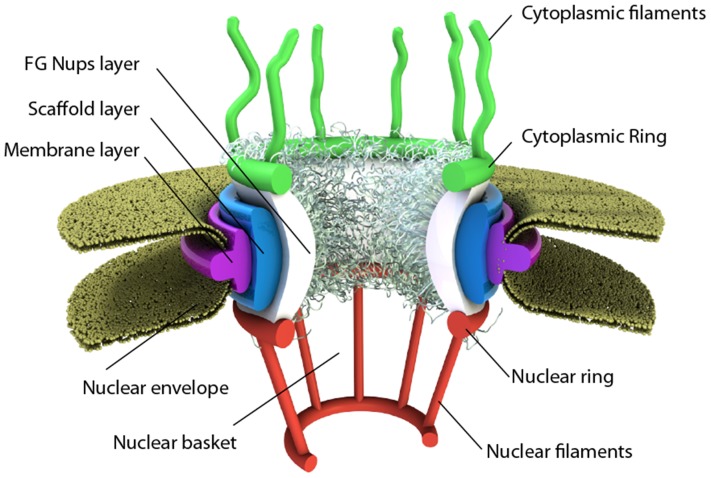
Schematic of the nuclear pore complex. The pore is anchored to the nuclear envelope by a membrane layer that surrounds the scaffold layer. This scaffold layer provides structure and serves as an anchor for Nups that contain both structured domains as well as highly unstructured domains that are thought to form a barrier that excludes non-interacting molecules while allowing for selective transport of others. This central channel exhibits eight-fold rotational symmetry and has eight cytoplasmic filaments as well as eight nuclear filaments protruding into the cytoplasm and nucleoplasm respectively. The nuclear filaments are bound via a ring, resulting in a basket structure.

The NPC itself is a ∼60 MDa (in yeast) to ∼125 MDa (in vertebrates) macromolecular assembly composed of multiple copies of ∼30 different proteins termed nucleoporins (Nups) that are evolutionarily conserved across eukaryotes and embedded in the nuclear membrane with eight-fold radial symmetry [1]–[4]. These Nups form eight cytoplasmic filaments that protrude from the nuclear envelope into the cytoplasm and another eight that project into the nucleus and are bound by a ring at their distal end to form a basket. The pore is anchored to the nuclear envelope by a membrane layer that surrounds the scaffold layer (Fig. 1). This scaffold layer provides structure and serves as an anchor for Nups that contain both structured domains as well as highly unstructured domains – rich in phenylalanine-glycine repeats –that are believed to be principally responsible for selective transport (FG-Nups). The FG-rich regions of these Nups present an affinity for hydrophobic patches present on transport receptor proteins involved in shuttling cargo across the nuclear envelope. The mechanism by which these Nups regulate transport remains a topic of much debate and has lead to the proposal of several competing models such as Brownian affinity gating [2], [5], [6], selective phase [7]–[11], affinity gradient [12], [13], reversible collapse [14]–[16] and reduction of dimensionality [17], among others. Additional details of NPC structure and function can be found in our recent review [18].

While these models aim to explain the pore's selectivity, other models were proposed to resolve contributors to transport directionality. The affinity gradient model emerged as a plausible explanation as a result of *in vitro* measurements in yeast and vertebrae demonstrating the presence of an increasing affinity gradient in Nups moving from the cytoplasm to the nucleus – suggesting that this affinity gradient provided transport directionality to cargo bound to transport receptors [12], [13]. Other studies found that the source of directionality of import complexes was a result of the steep RanGTP gradient present across the pore with high concentration of RanGTP in the nucleus, stemming from the presence of the nucleotide exchange factor RanGEF (Ran Guanine nucleotide Exchange Factor) and low concentrations of RanGTP in the cytoplasm due to hydrolysis via RanGAP (GTPase activating protein). These studies showed that reversal of the RanGTP gradient across the pore resulted in the reversal of transport directionality in spite of any affinity gradient [19]. Nevertheless, the contribution of the observed affinity gradient or a partial affinity gradient to transport efficiency remains unexplored with traditional experimental methods. More specifically, it is unclear whether (*i*) the presence of a Nup-Impβ affinity gradient affects nucleocytoplasmic transport rate and whether (*ii*) there exists an affinity gradient that can optimize transport rate beyond that of the reported *in vitro* gradient. To answer these questions, we have developed an agent based model to perform *in silico* measurements of Impβ translocation across the NPC.

### Agent Based Modeling

**Figure 2:**
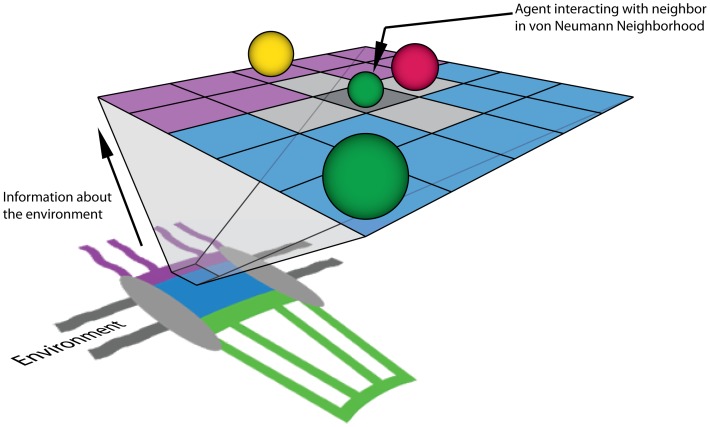
Simplified representation of the agent based model. Abstract cartoon representation of the nuclear pore structure environment (not to scale) projected onto a simplified, 2-dimensional, on-lattice ABM with agents representing proteins that move within the system and interact with other agents within their von-Neumann neighborhood. The actual model consists of a three-dimensional representation of the NPC structure and physiologically relevant concentrations of biochemical factors and channel dimensions. In our model, the purple region representing the cytoplasmic periphery is treated as a compartmentalized volume containing non-interacting Nup and Impβ-interacting FG-Nup agents. Similarly, central channel (blue) and nuclear basket (green) regions are represented by compartmentalized volumes, containing both non-interacting and interacting Nup agents at physiologically meaningful concentrations. Grey regions of the diagram represent the scaffold and nuclear envelope regions of the model that are impermeable to diffusing species.

Agent based modeling (ABM) is a robust computational technique used to simulate the spatiotemporal actions and interactions of real-world entities or “agents”, in an effort to extract their combined effect on the system as a whole. Both space and time can be discretized in an ABM, giving these autonomous agents the ability to move and interact with other agents and their environment at each timestep over a given duration. Simple behavioral rules govern the movement and interaction of each individual entity in an effort to reproduce or predict more complex behaviors of multiple entities. Such a model attempts to simulate the emergence of complex phenomena that may not be apparent when simply considering individual entities. Agent based modeling has seen applications in a broad range of fields ranging from artificial intelligence and gaming to modeling emergent social behavior such as the spread of disease and outcomes of financial markets [20]–[23]. In their simplest form, on-lattice agent based models consist of a mesh of “cells” that make up the discretized space that agents occupy. The agents occupy these cells and are typically only aware of other agents within their “neighborhood”; in the simplest form a neighborhood consists of adjacent cells. Agents are given the ability to move into adjacent cells and to interact with other agents with some probability in conjunction with governing rules that define what movement and interactions are possible (Fig. 2). On-lattice agent based models have previously been applied to biological systems involving diffusion, binding and unbinding [24]–[26]; establishing methods for event probability selection – relating diffusion and rate constants to event probability – will improve model accuracy and enable quantitative analysis of results from these models [27], [28].

In the present work we develop a method for relating real world rate constants to molecular binding and unbinding probabilities within the agent based model. We then build upon our ABM framework [27] to explore the role of an affinity gradient between Nups and the nuclear transport factor, Impβ in nucleocytoplasmic import efficiency. We model the system using affinity gradients derived from *in vitro* experiments and compare these to NPCs lacking affinity gradients as well as a wide range of forward and reverse affinity gradients in order to address the following questions: (*i*) Does the presence of a Nup-Impβ affinity gradient affect transport rate? (*ii*) Does there exist an affinity gradient that can optimize transport rate beyond that of the reported *in vitro* gradient?

To answer these questions, simulations were carried out using a computationally efficient, spatiotemporally detailed, three-dimensional agent-based model developed specifically for modeling molecular diffusion, binding and unbinding events with consideration for physical factors such as molecular crowding and steric repulsion. In addition to movement and interaction rules, event probabilities govern system dynamics in the agent-based model. Methods for accurate selection of movement, binding and unbinding probabilities to best represent actual diffusion coefficients and kinetic rate constants can build confidence in the output of agent based models and deductions from these models. The procedure for relating real world rate constants to molecular binding and unbinding probabilities is detailed in the Materials and Methods.

## Materials and Methods

### Probability Selection for Molecular Movement on an ABM Lattice

In our previous work we proposed a method for movement probability selection based on molecular diffusion coefficient along with algorithms for realistic consideration of crowding and steric repulsion [27] that was also used in the current model:
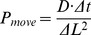
(1)


Here, movement probability of an agent is determined by its diffusion coefficient (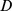), simulation timestep (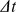) and lattice discretization length (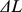). We implemented the *reduced probability (RP)* method to account for the steric effects of multiple agents occupying individual lattice sites [27].

### Probability Selection for Molecular Binding and Unbinding Events on an ABM Lattice

The simpler case of the molecular unbinding event, which is representative of a first-order unimolecular reaction, can be modeled in the ABM using an unbinding probability, for which derivation of the relationship between kinetic rate constant and probability is trivial. The reversible binding of two molecules *A* and *B* is given in Eq. (2), followed by the rate law for the unbinding event as a function of number of bound molecules within the volume of interest (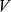):
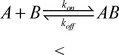
(2)

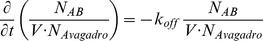
(3)


Change in the number of bound molecules (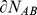) is a function of elapsed time (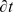), kinetic rate constant (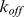) and initial number of bound molecules (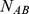) (Eq. (3)). Subsequently, the probability that two bound molecules become unbound is independent of interaction with other molecules; this unbinding probability (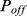) is shown in Eq. (6) in the limit of very small 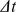.
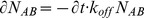
(4)

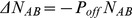
(5)

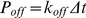
(6)


For molecular binding of two molecules, representative of a second order reaction between adjacent molecules on a lattice - factors such as number of lattice neighbors and lattice size must be considered. This relationship can be derived from the second order rate law as a function of number of unbound molecules within the volume of interest as shown in Eq. (7).
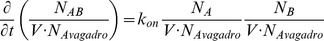
(7)


Similar to the case of unbinding, change in number of bound molecules can be expressed as a function of binding events in terms of number of unbound molecules (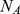 and 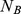) in the volume and their binding kinetic rate constant (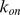) (Eq. (8)) as well as a function of binding probability (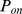) and the probability of finding neighboring binding molecules within the lattice system (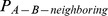), Eq. (9).
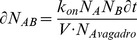
(8)

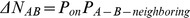
(9)


The likelihood of finding two unbound agents, *A* and *B*, neighboring each other on the lattice (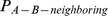) is a function of the number of unbound *A* molecules (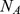), number of unbound *B* molecules (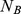), number of lattice cells (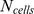), where number of lattice cells in the system is much larger than the number of unbound molecules and the number of lattice neighbors each cell has (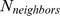), Eq. (10).
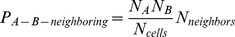
(10)


Subsequently, the probability of a binding event between two neighboring molecules (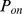) can be derived by solving for the likelihood of neighboring binding molecules in the system (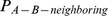) and combining with Eqs. (8) and (9) as shown in Eq. (11).
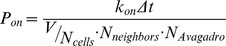
(11)


The general form of the probability of binding provided in Eq. (11) is valid for two molecules of different types. For binding events consisting of two molecules of the same type, the probability is reduced by half. Furthermore, Eq. (11) represents the case where the lattice is restricted to containing a single molecule per cell. In the case where multiple smaller molecules can occupy a single cell (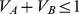), a correction factor (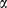) must be added to the number of neighbors since two unbound agents within the same cell can bind one another. Eq. (12) provides an approximation for the correction factor as a function of the sum of molecular volumes and cell volume.
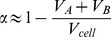
(12)


It should be noted that timestep selection is governed by the smallest of all time scales associated with diffusion or molecular interaction. In other words, the simulation timestep should be selected in a manner so that movement or binding/unbinding event probability does not exceed a value of one.

### Comparison of On-Lattice ABM Method to Deterministic ODE Solution

The agent based modeling framework used was an extension of the framework described in our previous publication, which explored methods for accounting for diffusion in agent based models of reaction-diffusion. The combined Reduced-Probability & Volume-Limit (RP+VL) method was used to govern diffusion behavior [27]. Binding events occurred between neighboring agents or agents within the same lattice point with probabilities as defined in Eq. (11), while unbinding events occurred with probabilities as defined in Eq. (6). Binding and unbinding rules were executed in random order for each agent type at each timestep to avoid the possibility of biasing a particular agent type to a specific bound or unbound state.

**Figure 3:**
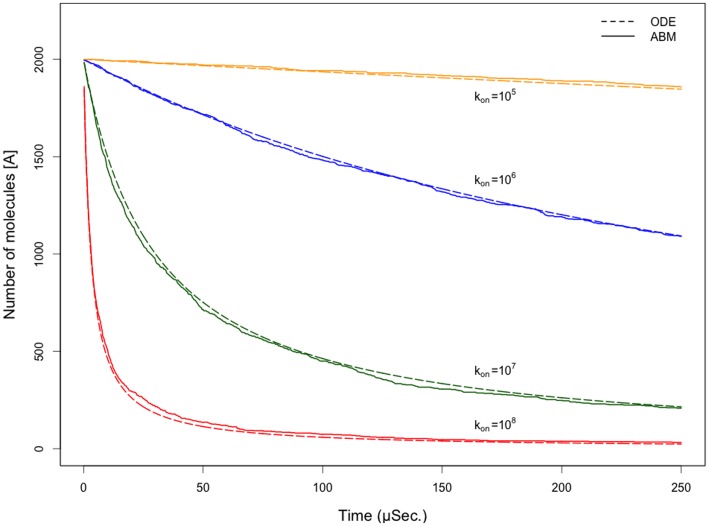
Comparison of ABM and ODE time course data. Comparison of time-course data from an agent based model of molecular binding to that of the numerical solution of the ordinary differential equation for the same event. Probability selection using the relationship in Eq. 11 produces similar behavior to that of the numerical solution in a well-mixed system at multiple rate constants with the addition of stochasticity that is expected from natural systems.

In order to validate these methods, we used the binding relation given in Eq. (11) to relate rate constants to event probabilities by modeling a system consisting of an initial concentration of 3 mM (2000 molecules in the well-mixed volume) molecules of type *A* that undergo a irreversible binding event, 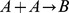. We compare the time-course data of the model using a deterministic ordinary differential equation (ODE) solver to that of the stochastic agent based model solution in Fig. 3 for multiple rate constants. The ABM solution reproduces the average behavior of the ODE solution without the unnatural smoothness that is seen in the deterministic model.

### ABM System and Simulation Details

The model environment consists of a 42,108 element, three-dimensional lattice comprised of elements with dimensions of 5 nm×5 nm×5 nm. The lattice size was selected to accommodate the volume associated with the Stokes radius of the largest single-agent species in the system, in this case Impβ (Nups being represented by a collection of multiple agents). Additionally, the model allowed for multiple agents of the same or different species type to occupy the same lattice element at any given time, so long as the available volume of a lattice element was not exceeded by agents diffusing into it. Discrete lattice elements belong to one of six region types, cytoplasmic, nuclear membrane, nucleoplasm, cytoplasmic filament periphery, central channel or nuclear basket. The cytoplasmic region contains Impβ molecules at a steady state concentration of 2.5 µM while the nucleoplasm contains RanGTP molecules at a steady state concentration of 1 µM throughout the simulation [29]. The 35 nm thick nuclear membrane which partitions the two compartments is impermeable to all agent types and contains a single nuclear pore with diameter of 30 nm at the center and 50 nm at the peripheries. The cytoplasmic filament periphery consists of a 50 nm diameter region that extends 30 nm into the cytoplasm while the nuclear basket is composed of a basket shaped region that extends 55 nm into the nucleoplasm [4], [30]. The cytoplasmic periphery, central channel and nuclear basket each contain 24, 80 and 32 agents respectively, which represent the distribution of FG Nups [31]. In addition to these FG agents, non-FG agents are added to the channel to represent regions of the Nups that lack affinity for Impβ but play a role in sterically repelling molecules, with the sum of the volume of these Nups corresponding to experimentally reported volumes [31]. Impβ and RanGTP agents are free to diffuse throughout the system while FG agents and non-FG agents are restricted to movement within their respective pore regions in order to maintain the permeability barrier. Impβ and RanGTP agents bind with an on rate of 9.6×10^4^µM^−1^sec^−1^ [29] while Impβ binds and unbinds FG-Nup agents with a dissociation constant that was varied from 200 nM to 2 mM [32].

For simulations where an influx rate was reported (units: Sec^−1^), the system was initially brought to steady state. Subsequently, an *in silico* microinjection of inert molecules ranging in size from a Stokes radius of 0.531 nm to 2.819 nm was administered for validation of the model's ability to reproduce the size exclusion properties of the nuclear pore as demonstrated in prior experiments [33]. In addition, the same microinjection was performed using Impβ to compare the model's influx rate for the karyopherin to what had been reported in experiments from other groups [29]. Simulations were run for a length of 25 to 75 seconds using a timestep of 2.5×10^−6^ seconds with 100 independent stochastic ABM simulations performed for each configuration. Time course concentration data for each configuration was averaged over the 100 independent simulations and data points were fitted to Eq. (13) for comparison with experimental values reported by Mohr and colleagues [33].
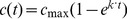
(13)


Where *c* represents concentration of microinjected species and *k* represents the influx rate of a given species into the nucleus, which follows first-order kinetics [33].

For simulations where Impβ transport rate was reported (units: molecules/Sec), simulations were run for a length of 2.5 seconds using a timestep of 2.5×10^−6^ seconds. The system was allowed to reach steady state in the first 1 second and linear regression was performed on the remaining 1.5 seconds of simulation data to quantify the number of Impβ transported to the nucleoplasm as a function of time. The transport rate for each set of 100 simulations was averaged and reported along with standard error.

### Importinβ Multivalency and Nup-Impβ Affinity

The transport receptor Impβ has been shown to contain multiple hydrophobic patches that serve as FG binding sites and are believed to play a critical role in the shuttling of Impβ and cargo transport [34]. It has been shown experimentally that Impβ contains two FG binding sites near the N terminus (between HEAT repeats 5 and 6, another between HEAT repeats 6 and 7) as well as two FG binding sites near the C terminus (between HEAT repeats 14 and 15, another between HEAT repeats 15 and 16) [35]–[37]. Furthermore, Isgro and Schulten identified up to six additional FG binding sites on Impβ using computational methods [38]. Subsequently, experimentally determined macroscopic affinities (or multivalent affinities) are the result of combined microscopic affinities (or monovalent affinities) between FG repeats and the multiple binding sites on Impβ [32].

In our agent-based model, FG-Nups and Impβ bind and unbind with a single probability that corresponds to the macroscopic affinity and represents the combined effect of microscopic affinities – a simplification that is common to other models with coarse granularity [39]–[41]. This simplification is further justified when considering that: (*i*) the high concentration of FG repeats that a Nup presents to Impβ (∼150 mM) results in the receptor strongly tending toward the fully bound state [32], and (*ii*) consideration that the on-rate for association of Impβ with FG-Nups has been approximated to be 10^7^–10^8^ M^−1^s^−1^ [12], which suggests that the receptor reaches a fully bound state within the span of the timestep used in our simulations (2.5×10^−6^ seconds). Finally, coarse-grained Brownian dynamics simulations of cargo transport through the pore have confirmed that once Impβ is hydrophobically engaged with the pore, its monovalent binding sites become fully saturated at the timescales used in this agent based model [42], [43].

### Model Validation

**Figure 4:**
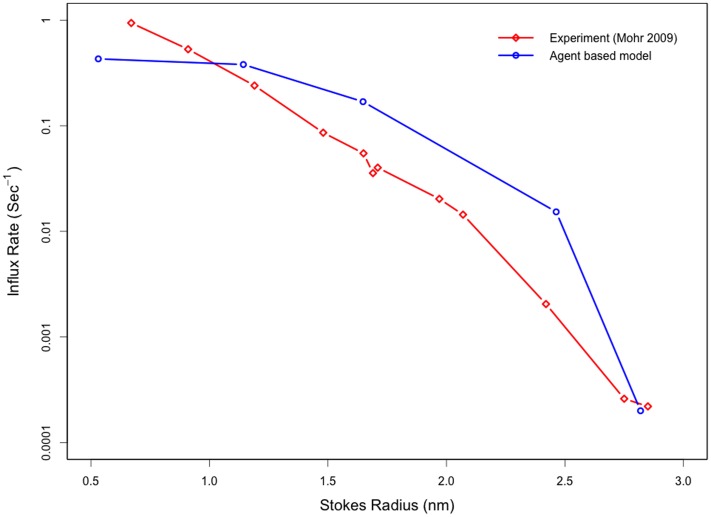
The agent based model recapitulates the experimentally observed size-dependent permeabilities of passive cargos through the nuclear pore. Following a simulated microinjection of non-interacting species in the cytoplasm, the *in silico* pore is observed to inhibit the influx of larger species while allowing smaller species to diffuse through the pore. This is in agreement with previous experimental observations.

*In silico* experiments were performed to determine the model's ability to recapitulate experimentally-determined, size-dependent permeabilities for passive cargos as well as for Impβ [29], [33]. These simulations served as a control to validate the model and associated algorithms' ability to simulate selective transport. Following a simulated microinjection of non-interacting species in the cytoplasm, the *in silico* pore is observed to inhibit the influx of larger species while allowing smaller species to diffuse through the pore (shown in Fig. 4). Influx rates of non-interacting species with Stokes radii of ∼1 nm are on the order of 0.1 s^−1^ while larger species with Stokes radii of >2.5 nm have influx rates of less than 0.001 s^−1^. As expected, a reduced influx rate was not observed for larger species that had affinity for the FG-Nups. To test this behavior, experiments similar to those performed for non-interacting species were repeated, replacing the non-interacting species with 2.5 µM labeled Impβ – in addition to the steady-state concentration of unlabeled Impβ. Influx of Impβ into the nucleus was measured for 100 simulations, averaged and fit to Eq. 13. Influx rates of 0.367 s^−1^ and 0.391 s^−1^ were observed for a pore with uniform Nup-Impβ affinity of 200 µM and a pore with the optimal Nup-Impβ affinity gradient respectively. These values are comparable with experimentally measured influx rates of 0.4 s^−1^ for Impβ [29].

Finally, the model's sensitivity to various simulation parameters was explored. Previous studies have proposed that import receptors are the rate limiting species in the import pathway [44], [45]. Using the model, we observed a ∼50% increase in Impβ shuttling when cytoplasmic Impβ steady-state concentration was increased two-fold. Conversely, there was no significant increase in Impβ shuttling as nuclear RanGTP concentrations were increased up to ten-fold. These findings are discussed in further detail below. In addition to assessing sensitivity to biochemical species, we assessed the model's sensitivity to the simulation timestep used. Cutting the simulation timestep in half (from 2.5×10^−6^ seconds to 1.25×10^−6^ seconds) resulted in an average change in absolute Impβ shuttling rates of 5.5±3.4% for a range of Nup-Impβ affinities. Nevertheless, the overall relationship between Nup-Impβ affinity and Impβ transport rate were preserved, regardless of the selected timestep.

## Results and Discussion

### Transport in the Absence of an Affinity Gradient

**Figure 5:**
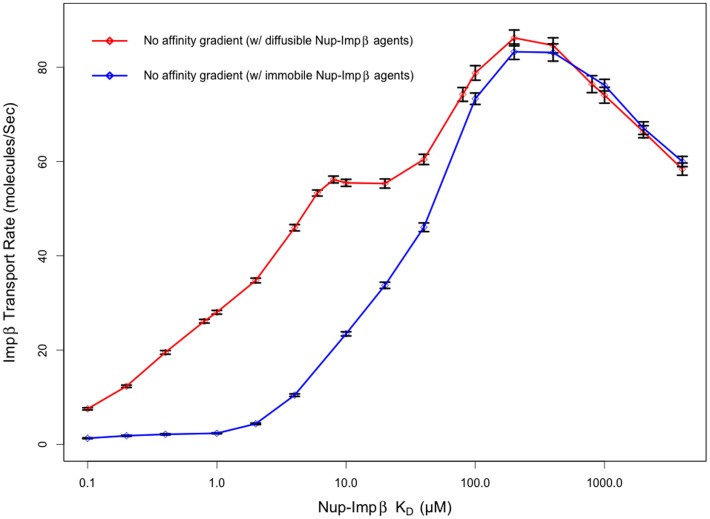
Impβ transport rate through a pore with Nups of uniform affinity. Transport rates for ABM simulations of Impβ through a nuclear pore containing Nups with uniform affinity (no gradient). Nup-Impβ affinity is varied from 100 nM to 4 mM. The transport rate exhibits biphasic behavior as a function of affinity. At very high affinities (low K_D_), Impβ is tightly bound to Nups, resulting in slow transport rates as the Nups become saturated. At very low affinities, Impβ isn't able to bind Nups as efficiently, reducing its resident time at the pore periphery and subsequently excluding it from the pore interior as a result of steric effects. Peak transport of 86.24±1.68 transports per second were observed at a Nup-Impβ affinity of 200 µM. Pores containing Nup bound Impβ agents that are capable of diffusing locally exhibit increased transport rate compared to simulation configurations where Impβ becomes immobile once bound to an FG-Nup.

Initially, Nup-Impβ affinity was kept homogenous throughout the NPC's three regions – cytoplasmic periphery, central channel and nuclear basket – to investigate how a uniform binding affinity affects transport rates. The results of these simulations display a biphasic behavior in transport rate as channel affinity is increased from 100 nM to 4 mM, with peak transport rate of 86.241±1.68 Impβ translocations per second observed for a channel with a dissociation constant of 200 µM (Fig. 5). Recent Brownian dynamics models of single-cargo transport have shown a similar biphasic behavior in transport time as a function of Nup-cargo-complex affinity [42]. It is worth mentioning that since Impβ is the only transportin considered in this model, relative transport rates are of more interest than absolute transport rates (absolute transport rates of Impβ may differ when considering the effects of competing transport receptors).

**Figure 6:**
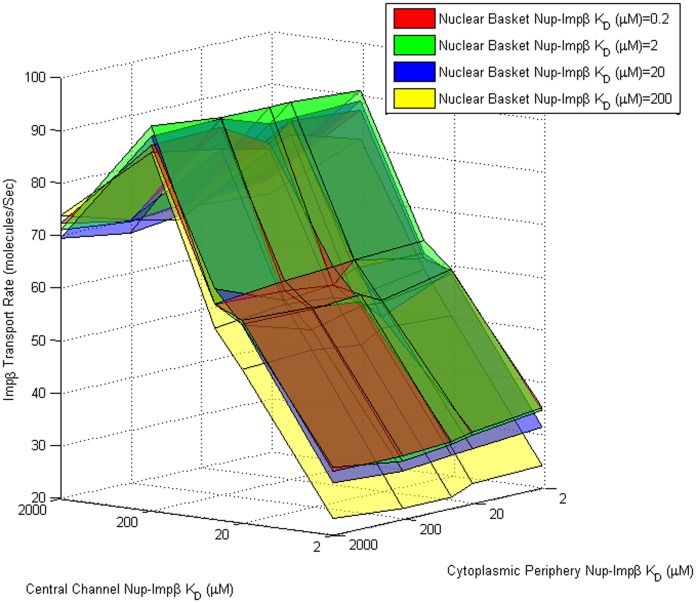
Transport rate as a function of cytoplasmic, central channel, and nuclear basket Nup-Impβ affinity. Impβ transport rate (z-axis) as a function of cytoplasmic (x-axis) and central channel (y-axis) Nup-Impβ affinity ranging from 2 µM to 2 mM. The four three-dimensional surfaces represent a range of nuclear basket affinities ranging from 0.2 µM to 200 µM. Transport rates appear to be least sensitive to cytoplasmic affinities and most sensitive to central channel and nuclear basket affinities. Varying central channel affinities results in biphasic behavior with maximum transport at K_D_≈200 µM. Transport rates appear to increase as nuclear basket affinity is increased up to K_D_≈10 µM and don't appear to show significant increase at higher affinities.

**Figure 7:**
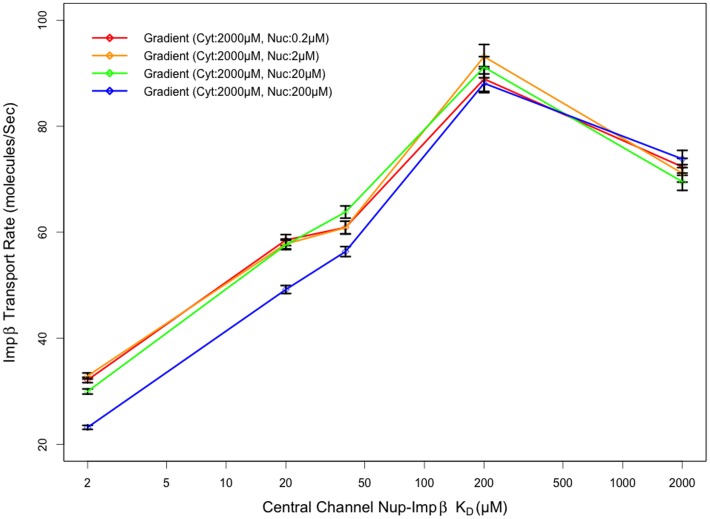
Transport rate as a function of central channel affinity. Impβ transport rate appears most sensitive to central channel affinity, regardless of nuclear basket affinity, with a peak transport rate when Nup-Impβ affinities are on the order of 100 µM. (Cyt: Cytoplasmic periphery, Nuc: Nuclear basket)

The presence of an optimal binding affinity for transport through a uniform channel is not surprising. As expected, very low dissociation constants will result in increased binding time between molecules, resulting in reduced mobility. Conversely, high dissociation constants will result in a reduction in the time that Impβ is bound to the channel, similar to a molecule with no binding affinity, relying on diffusion alone to traverse the Nup-obstructed channel. It was observed that the optimal dissociation constant of 200 µM for the uniform affinity pore was the same as the optimal Nup-Impβ affinity within the central channel of the pore with affinity gradient (Fig. 6 and Fig. 7). Coincidentally, this is also the region with the highest relative FG motif density [31].

The saddle point observed in the plot of Impβ transport rate as a function of Nup-Impβ affinity is due to the motility of Impβ when bound to high affinity Nups (Fig. 5 – No affinity gradient w/diffusible Nup-Impβ agents). To confirm this, we repeated the simulations but configured the system so that once an Impβ agent bound to an FG-Nup agent, the complex had a movement probability of zero. In this configuration, the Impβ transport rate decreased sharply as Nup-Impβ affinity was increased past 100 µM (Fig. 5 – No affinity gradient w/immobile Nup-Impβ agents). Our assumption that Nup-Impβ complexes are locally diffusive is based on the presence of hydrophobic FG-pockets, which bind Impβ on unstructured regions of the Nups. Furthermore, Brownian dynamics models demonstrate that cargo complexes continue to exhibit diffusive movement when bound to Nups lining the nuclear pore, albeit at a lower rate than their unbound state [42]. The combination of an increase in time bound between Nup and Impβ at higher affinities and the complex's local mobility leads to an increase in likelihood that the Impβ overcomes the Nup-dense central channel. However, the motility of Nup-bound Impβ agents has no effect on our primary parameter of interest – the Nup-Impβ binding affinity at which peak transport is observed.

### Simulation-Derived Transport Rates for Experimentally Measured Affinity Gradients

The model lacking an affinity gradient from the previous section was subsequently modified so that the Nup-Impβ affinity in each region matched *in vitro* reported affinities from two previous experiments as outlined in Table 1.

**Table 1:** Summary of affinity gradients and *in silico* derived transport rates.

Source	Cytoplasmic Nup Affinity (µM)	Central Channel Nup Affinity (µM)	Nuclear Basket Nup Affinity (µM)	*in silico* Transport Rate (Sec^-1^)	Ref.
**Yeast**	1.5	0.2	0.01	12.23±0.27	[13]
**Vertebrates**	0.2	0.1	0.01	7.58±0.21	[12]
**Model Optimum**	2000	200	10	94.73±1.92	

Summary of *in vitro* affinity gradients for yeast and vertebrates and the agent based model derived transport rate corresponding to each affinity gradient. The model optimum affinity gradient is included for comparison. The ratio of gradients between yeast and model optimum are comparable while the magnitude of the individual affinities is approximately 1000 times weaker in the model optimum.

Using affinity gradients measured *in* vitro in yeast and vertebrates, our model predicts transport rates that are approximately an order of magnitude lower than the peak transport rate observed for pores with uniform 200 µM affinity. The presence of this affinity gradient brought about minimal gains in transport rate when compared to pores lacking a Nup-Impβ affinity gradient in the same nano-molar affinity range. Low transport rates at these experimentally derived affinities can be attributed to the very slow off-rate between Impβ and Nups that act to hinder transport and are in contrast to simulations where significantly higher transport rates are seen in pores containing Nups with micro- to milli-molar affinities. It has previously been suggested that affinities derived from *in vitro* experiments are too tight to account for experimentally observed transport rates, with the simplest reason being that the associated off-rates are much slower than the observed transport times of *in vivo* cargo [10], [44], [46], [47]. More recently, it has been shown that *in vivo* affinities between Nup and Impβ are likely much lower than *in vitro* measurements claim due to the presence of non-specific competitors in the cell milieu which are generally not considered in *in vitro* studies [32], [48], [49].

### Nucleocytoplasmic Transport Sensitivity to Pore Affinity Gradient

To quantify the contribution of each region's affinity to Impβ transport rate, we varied the *in silico* affinity of FG-Nup agents for Impβ in each of the three regions independently. The range of affinities explored spanned 2 µM to 2 mM in the cytoplasmic periphery and central channel and 200 nM to 200 µM in the nuclear basket (Fig. 6). This range allowed us to explore transport rates for moderate and steep gradients in both forward and reverse affinity gradients.

**Figure 8:**
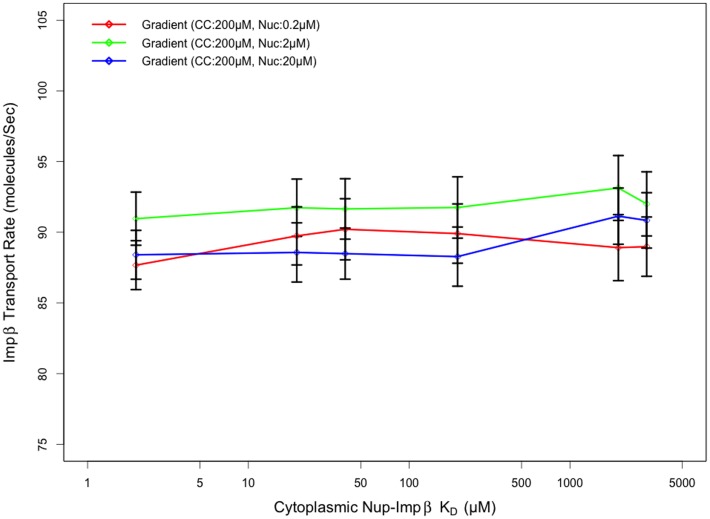
Transport rate as a function of cytoplasmic affinity. Transport rate appears insensitive to cytoplasmic Nup-Impβ affinity as opposed to central channel and nuclear basket Nup-Impβ affinity. An increase or decrease to affinity in the cytoplasmic region by an order of magnitude results in a change in transport rate that is within a standard error. (CC: Central Channel, Nuc: Nuclear basket)

As our results indicate, the transport rate appears mostly insensitive to the affinity of FG-Nups in the cytoplasmic periphery for Impβ, especially when compared to that of the other two regions. Varying the affinity of FG-Nups for Impβ in the central channel of the pore resulted in a clear biphasic behavior in transport rates observed, with very low and high affinity Nups hindering the transport of Impβ irrespective of affinity in the other two regions. The contrast between the effects of affinity in the cytoplasmic region compared to the central channel can likely be attributed to the difference in Nup density in each region; the higher Nup concentration within the central channel [31] makes affinity a much more critical parameter. Very low affinities prevent Impβ from binding FG agents and traversing the channel as Impβ molecules are sterically repelled by the Nups – while very high affinities result in longer binding and reduced mobility of Impβ molecules. Our data suggest that a FG-Impβ K_D_ of 200 µM in the central channel is ideal for transport irrespective of cytoplasmic and nuclear basket affinities. Transport rates were observed to peak at cytoplasmic FG-Impβ K_D_ of 2 mM, although this was not much higher (less than one standard error) than transport rates observed with K_D_ ranging from 2 µM to 4 mM. The relationship between cytoplasmic periphery Nup-Impβ affinity and transport rate is depicted in more detail in Fig. 8.

**Figure 9:**
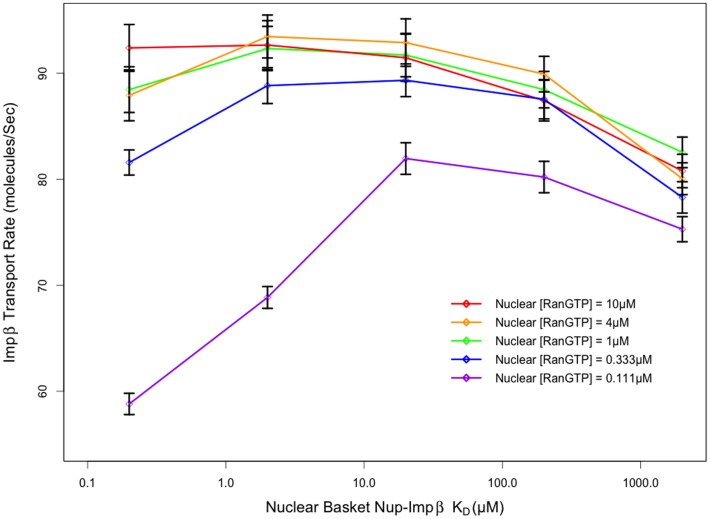
Transport rate as a function of nuclear basket affinity. Transport rates are very sensitive to nuclear basket Nup-Impβ affinity, with maximum transport rates emerging in the presence of a high affinity target for Impβ in the nuclear basket. Transport rates peak at an affinity of ∼2 µM with a slight decrease in transport rate as affinities are increased beyond that. This peak in transport rate doesn't appear to be due to a lack of RanGTP to terminate transport at the nuclear periphery of the pore since there aren't significant changes to transport rate under very high nuclear RanGTP concentrations. Conversely, when nuclear RanGTP concentrations are much lower than physiological values, the effect on transport rate is more noticeable.

Affinity of nuclear basket FG-Nups for Impβ also plays a critical role in determining transport rate. Our model indicates that as this affinity is increased, the transport rate is also observed to increase up to a limit that is dependent on the availability of RanGTP. As shown in Fig. 9, for the standard case of nuclear RanGTP concentration of 1 µM, the transport of Impβ increases as nuclear basket affinity is increased, up to an affinity of 2 µM. A decrease in Impβ transport rates is observed at affinities higher than 2 µM (lower K_D_), under standard RanGTP concentrations. This observation suggests that at low affinities, Impβ is loosely bound to the pore and subsequently has a low residence time in the basket, free to diffuse back to the central channel or into the nucleoplasm where it can interact with RanGTP. As affinity is increased, Impβ residence time in the basket increases, preventing it from diffusing back into the central channel and increasing the likelihood of interaction with RanGTP and subsequent release into the nucleoplasm. At very high affinities >2 µM, Impβ appears to become “stuck” at the entry to the nuclear basket, reducing the likelihood of interaction with RanGTP and decreasing the overall rate of Impβ shuttling.

When nuclear RanGTP concentrations were increased, up to ten-fold, the absolute transport rate was not observed to change significantly. Conversely, when Impβ concentrations were increased by as little as two-fold, there was a consistent ∼50% increase in Impβ shuttling into the nucleus observed across all nuclear basket affinity configurations. This is in agreement with previous studies that propose that import receptors, rather than Ran, are the limiting species in the import pathway [44], [45].

Although an increase in nuclear RanGTP concentration had little effect on Impβ shuttling, a decrease in nuclear RanGTP concentration was shown to reduce Impβ shuttling from cytoplasm to nucleus. Nevertheless, a nearly ten-fold decrease in nuclear RanGTP concentration resulted in a minimum of ∼11% decrease in Impβ shuttling across the nuclear basket and a maximum decrease of ∼37% at the lower 0.2 µM affinity as shown in Fig. 9 – suggesting that import is more sensitive to the availability of transport receptors than the availability of Ran. The wide spread in Impβ transport rates as a function of RanGTP concentration at high affinities compared to that at lower affinities is due to the significance of RanGTP in each scenario. At low affinities, Impβ is loosely bound to the pore and is free to diffuse back to the central channel or into the nucleoplasm and is subsequently insensitive to RanGTP concentration. At very high affinities >2 µM, Impβ appears to become “stuck” at the entry to the nuclear basket and a higher concentration of RanGTP is required to release RanGTP. At this high affinity and in the absence of sufficient RanGTP, Impβ transport decreases significantly as it is less likely to spontaneously release from the pore.

Our results suggest that the peak transport rate for Impβ shuttling are produced with a Nup-Impβ affinity gradient consisting of 2000 µM in the cytoplasmic fibril region, 200 µM in the central channel and 10 µM in the nuclear basket which produced a transport rate of 94.73±1.92 transports per second. This produces a regional Nup-Impβ affinity ratio of 1∶20∶200 (nuclear: central channel: cytoplasmic). Despite the difference in magnitude, the *in vitro* measurements of yeast affinity gradient exhibit a similar affinity gradient ratio of 1∶20∶150. It would be speculative to state that competitors in the cellular milieu would decrease *in vitro* affinities in a linear manner that would result in µM range affinities with the same gradient ratio. However, recent experiments by the Rout group showing the effective Kd after the addition of 0.1 mg/mL of lysate as competitor hint at such a relationship [32]. Furthermore, the difference between the *in vitro* affinity gradient ratio and the *in silico* derived optimum is restricted to the cytoplasmic affinity (150 vs 200 respectively) which, as stated previously, is the least significant determinant of overall transport rate among the three regions. Simulation using a 1500 µM cytoplasmic affinity instead of the optimum 2000 µM to reproduce the affinity gradient ratio seen *in vitro* resulted in less than a 1% reduction in overall transport rate.

Finally, as indicated by these simulations, transport rates are relatively insensitive to the Nup-Impβ affinity in the cytoplasm. Varying the cytoplasmic affinity from the optimum 2000 µM to 200 µM, eliminating the steep gradient between the cytoplasmic and central channel Nups, results in only a 2% decrease in transport rate, well within the standard error of our measurements. As previously mentioned, this is not the case for central channel and nuclear basket Nups. This observation supports the notion that a continuous affinity gradient isn't necessary for efficient transport [32]; rather, the majority of the pore can be composed of Nups with a moderate affinity for Impβ (K_D_≈200 µM) combined with high affinity Nups in the nuclear basket (K_D_≈10 µM) to achieve transport rates comparable to computationally derived optimum values.

Interestingly, the idea of maximizing transport rate by inhibiting diffusion at a terminal side of the pore is not limited to the Impβ import pathway. Hydrogels composed of Nup214 and Nup358 (which are located on the farthest cytoplasmic side of the pore) have been shown to selectively inhibit the diffusion of CRM1, an export receptor of the same karyopherin-β family as Impβ, while allowing the import receptor Impβ to diffuse across rapidly [50], [51]. Such a high affinity target for export receptors at the cytoplasmic side of the pore and likewise, as our model shows, a high affinity target for import receptors at the nuclear side of the pore can improve transport efficiency by reducing backflow of export and import complexes, and increasing the likelihood that they interact with RanGAP and RanGTP respectively.

## Conclusions

The presence of an affinity gradient, or at the very least, a high affinity target within the nuclear basket for import cargo complexes has been observed but remains contested. The effect of such affinity gradients on transport rates was not previously explored in detail. We developed a coarse-grained, biophysical model of the nuclear pore complex translocating Impβ under various forward and reverse affinity gradients. Our results are in agreement with previous reports that the affinity gradient within the nuclear pore is not essential for cargo transport [2], [52]; rather an affinity gradient, or more specifically, a high affinity target within the nuclear basket can increase overall transport rates. In fact, the reversal of the affinity gradient, shown in Fig. 6, illustrates that net movement of the Impβ molecules continues in the same direction, albeit at a lower rate, indicating that the presence of a RanGTP concentration gradient is more influential than the contribution of an affinity gradient. Nevertheless, our results reveal that the slope of the affinity gradient that maximizes transport through the pore is very similar to the slope of the affinity gradient measured *in vitro*, albeit at much lower affinity values ( µM vs. nM). These lower affinity values are in agreement with the range of affinities reported in recent experimental findings, suggesting that competitors present *in vivo* reduce the effective affinity gradient. These findings could have additional implications for the design and optimization of highly efficient artificial nanopores. These modeling techniques can be used to further assess the role of nucleoporin density and distribution along with channel geometry on transport efficiency and selectivity in an effort to optimize the design and function of artificial nanopores.
